# Extended treatment of Cushing’s disease with pasireotide: results from a 2-year, Phase II study

**DOI:** 10.1007/s11102-013-0503-3

**Published:** 2013-08-14

**Authors:** M. Boscaro, J. Bertherat, J. Findling, M. Fleseriu, A. B. Atkinson, S. Petersenn, J. Schopohl, P. Snyder, G. Hughes, A. Trovato, K. Hu, M. Maldonado, B. M. K. Biller

**Affiliations:** 1 Division of Endocrinology, Polytechnic University of Marche, 60126 Ancona, Italy; 2 INSERM U1016, Institut Cochin, Paris, France; 4 Reference Centre for Rare Adrenal Diseases, Department of Endocrinology, Assistance Publique Hôpitaux de Paris, Hôpital Cochin, Paris, France; 5 University Paris Descartes, Paris, France; 6 Division of Endocrinology, Metabolism, and Clinical Nutrition, Medical College of Wisconsin, Milwaukee, WI USA; 7 Departments of Medicine and Neurological Surgery, Northwest Pituitary Center, Oregon Health & Science University, Portland, OR USA; 8 Regional Centre for Endocrinology and Diabetes, Royal Victoria Hospital, Belfast, UK; 9 ENDOC Center for Endocrine Tumors, Hamburg, Germany; 10 Medizinische Klinik IV, Ludwig-Maximilians University, Munich, Germany; 11 Penn Pituitary Center, University of Pennsylvania, Philadelphia, PA USA; 12 Clinical Development, Oncology Business Unit, Novartis Pharma AG, Basel, Switzerland; 13 Clinical Development, Oncology Business Unit, Novartis Pharmaceuticals Corporation, Florham Park, NJ USA; 14 Oncology Biometrics and Data Management, Novartis Pharmaceuticals Corporation, Florham Park, NJ USA; 15 Neuroendocrine Clinical Center, Massachusetts General Hospital, Boston, MA USA

**Keywords:** Pasireotide, SOM230, Cushing’s disease, Somatostatin analogue, Clinical trial

## Abstract

In a previous 15-day, Phase II study of patients with de novo or persistent/recurrent Cushing’s disease (core study), treatment with pasireotide 600 μg sc bid reduced urinary free cortisol (UFC) levels in 76 % of patients and normalized UFC in 17 %. The objective of this study was to evaluate the efficacy and safety of extended treatment with pasireotide. This was a planned, open-ended, single-arm, multicenter extension study (primary endpoint: 6 months). Patients aged ≥18 years with Cushing’s disease who completed the core study could enter the extension if they achieved UFC normalization at core study end and/or obtained significant clinical benefit. Of the 38 patients who completed the core study, 19 entered the extension and 18 were included in the efficacy analyses (three responders, 11 reducers, four non-reducers in the core study). At data cut-off, median treatment duration in the extension was 9.7 months (range: 2 months to 4.8 years). At extension month 6, 56 % of the 18 patients had lower UFC than at core baseline and 22 % had normalized UFC. Of the four patients who remained on study drug at month 24, one had normalized UFC. Reductions in serum cortisol, plasma adrenocorticotropic hormone, body weight and diastolic blood pressure were observed. The most common adverse events were mild-to-moderate gastrointestinal disorders and hyperglycemia. Pasireotide offers a tumor-directed medical therapy that may be effective for the extended treatment of some patients with Cushing’s disease.

**Electronic supplementary material:**

The online version of this article (doi:10.1007/s11102-013-0503-3) contains supplementary material, which is available to authorized users.

## Introduction

Cushing’s disease is the clinical consequence of chronic hypercortisolism caused by an adrenocorticotropic hormone (ACTH)-secreting pituitary adenoma [1]. Patients with Cushing’s disease have 4.8-fold higher mortality than the general population [2] and a high level of cardiovascular, metabolic and osteoporotic comorbidities [2–4]. First-line treatment for patients with Cushing’s disease is transsphenoidal surgery, with remission rates of 65–90 % in patients with a microadenoma, if performed by an expert pituitary surgeon [1]. Second-line options include repeat surgery, radiotherapy, medical therapy and bilateral adrenalectomy [1]. However, lower success rates are seen after repeat surgery than after the initial surgery, and hypopituitarism is more common [1]. Hypopituitarism is also common after radiotherapy [5], whereas bilateral adrenalectomy results in permanent hypoadrenalism [1].

The most commonly used medical therapies are steroidogenesis inhibitors, none of which have been evaluated in large, prospective clinical trials. These agents are generally used off-label [1, 6]. Recently, pasireotide has been approved as a medical therapy for Cushing’s disease in the EU and US [7], and mifepristone is available to treat hyperglycemia associated with hypercortisolism in the US [8].

Corticotroph adenomas express multiple somatostatin receptor subtypes (sst), making somatostatin analogues a rational therapeutic option for patients with these tumors. Pasireotide is a multireceptor-targeted somatostatin analogue with high affinity for sst_1_, sst_2_ and sst_3_, and highest affinity for sst_5_ [9]. The receptor sst_5_ is present on most corticotroph adenomas [10]. In a 15-day, proof-of-concept, Phase II study in patients with de novo or persistent/recurrent Cushing’s disease, treatment with pasireotide 600 μg sc bid reduced urinary free cortisol (UFC) levels in 76 % (22/29) of patients and led to normal UFC levels in five patients (17 %) [11]. This paper presents results from the extension phase of the Phase II study and evaluates the long-term efficacy and safety of pasireotide in patients who chose to continue the medication.

## Patients and methods

### Patients

Patients with Cushing’s disease (aged ≥18 years) who had completed the 15-day, proof-of-concept, Phase II core study [11] were eligible to enter this extension phase if they had normal 24-h UFC levels at the end of the core study and/or, in the opinion of the investigator, obtained significant clinical benefit with pasireotide. Confirmation of Cushing’s disease was previously described in the core study [11]. Inclusion and exclusion criteria for the extension study are reported in the Supplementary Material.

This study was approved by the Independent Ethics Committee, Institutional Review Board or Research Ethics Board for each study center. The study was conducted according to the ethical principles of the Declaration of Helsinki. All patients provided written informed consent.

### Study design

This open-ended, single-arm extension study (ClinicalTrials.gov NCT00171951) was a planned extension to the 15-day core study [11]. Patients who achieved normalized UFC levels at the end of the core study continued at a dose of 600 μg sc bid. If UFC levels increased, the pasireotide dose could be increased to 900 μg sc bid. The dose of pasireotide could be reduced by 150 μg per injection at any time if the investigator believed that a drug-related adverse event (AE) was present.

Patients were to be discontinued from the extension study if they failed to demonstrate ongoing treatment benefit, experienced an unacceptable AE (defined as drug-related toxicity of Common Terminology Criteria [CTCAE v3.0] [12] grade 3 or 4 that did not resolve to grade 1 within 24 h), experienced evidence of disease progression or received any other therapeutic intervention for Cushing’s disease (see Supplementary Material for further details).

### Endpoints

The primary efficacy outcome was the proportion of patients with normalized UFC levels after 6 months of treatment (normal UFC range: 55–276 nmol/24 h; 20–100 μg/24 h). Secondary objectives included assessment of the safety and tolerability of multiple doses of pasireotide and trough plasma concentrations of pasireotide after chronic dosing.

### Assessments

Patients were classified as ‘UFC responders’ if their mean UFC level at month 6 was in the normal range; as ‘UFC reducers’ if their mean UFC level at month 6 was lower than that at core study baseline but not within normal limits; and as ‘UFC non-reducers’ if their mean UFC level at month 6 was equal to or higher than that at core study baseline or if the mean UFC level at month 6 was missing. Patients who discontinued prior to month 6 were classified as non-responders.

Blood samples for serum cortisol, plasma ACTH and pharmacokinetic assessments were collected before the morning pasireotide dose on study visit days. UFC assays were conducted in two central laboratories, one in Europe and the other in the US. Serum cortisol and plasma ACTH assays were assessed centrally by one laboratory (see Supplementary Material for further details).

Safety was assessed by recording all AEs, as well as by regular monitoring of vital signs, physical condition, body weight, hematology and blood chemistry. HbA_1c_ levels were recorded at extension baseline and then every 16 weeks. HbA_1c_ categories were: <upper limit of normal (ULN; normal defined as <5.7 %), between the ULN and 7 %, between 7 and 9 %, and over 9 %.

### Statistical methods

Summary statistics were provided for the primary and secondary endpoints. No formal statistical comparisons were performed for this study because of the small sample size. The analysis populations were defined as follows: the intention-to-treat (ITT) and safety populations consisted of patients who received at least one pasireotide dose in the extension period. The primary efficacy population consisted of those ITT patients whose mean UFC at core baseline, based on at least two UFC samples, was >ULN.

## Results

Of 38 patients who completed 15 days’ pasireotide therapy, 19 entered this extension phase, 17 women and two men, all Caucasian, mean age 43 years (range 22–73, standard deviation [SD]: 11.6). One patient had normal UFC levels on entry into the core study and was therefore excluded from the primary efficacy analysis. Three patients who entered the extension study had normal UFC levels at the end of the core study. Between completing the core study and entering the extension, 10 patients experienced a treatment interval. During this interim period, patients did not receive pasireotide (range of interval: 6–367 days). Information on other treatments for Cushing’s disease was not collected during the treatment intervals (see Supplementary Material for further details).

All patients entered the extension phase on a dose of 600 μg sc bid. At data cut-off, the treatment duration ranged from 2 months to 4.8 years and the median treatment duration was 9.7 months at that time. Three patients were continuing to receive treatment at the time of the data cut-off. Overall, 16 patients discontinued treatment during the extension study. The main reasons for discontinuation were to start new therapy for Cushing’s disease (five [26.3 %]), withdrawal of consent (three [15.8 %]), unsatisfactory therapeutic effect after 5–10 months (three [15.8 %]), AE (one [5.3 %]: new-onset type 2 diabetes mellitus), and abnormal laboratory value (two [10.5 %]: one increased HbA_1c_ and glucose and one elevated UFC). Seven patients discontinued treatment prior to month 6.

### Effects of pasireotide therapy at month 6

#### UFC levels at month 6

Of the 18 patients included in the primary efficacy analysis, four had normal UFC levels at month 6 (responders, 22.2 %; 95 % confidence interval [CI] 6.4–47.6). Six patients (33.3 %) achieved a reduction in mean UFC, but not to within normal range (reducers). One patient had a mean UFC level greater than at baseline and was classified as a non-reducer; the remaining seven patients were also considered to be non-reducers because they discontinued treatment prior to month 6.

In the 18 patients included in the primary efficacy analyses, mean UFC (± SD) decreased from core baseline by −802 ± 819 nmol/24 h to month 6 of the extension (Table 1).

**Table 1:** Change in UFC, serum cortisol and plasma ACTH from core baseline to day 15 and months 6, 12 and 24

	Core baseline	Day 15	Month 6	Month 12	Month 24
*UFC* (nmol/24 h) [μg/24 h]					
n^a^	17	17	11	4	4
Mean ± SD	1,220 ± 753 [442 ± 273]	522 ± 302 [189 ± 109]	421 ± 340 [153 ± 123]	398 ± 220 [144 ± 80]	514 ± 456 [186 ± 165]
Range	291, 2,760 [105, 1,000]	158, 1,133 [57.3, 411]	159, 1,267 [58.0, 459.1]	198, 707 [72, 256]	80, 1,141 [29.0, 413]
Mean change from core baseline ± SD	–	−711 ± 741^b^ [−258 ± 269]	−802 ± 819^c^ [−291 ± 297]	−1,202 ± 1,084 [−436 ± 393]	−1,241 ± 926 [−450 ± 336]
Range		−2,340, 367 [−848, 133]	−2,388, 34 [865, 12.3]	−2,367, −272 [−858, −99.0]	−2,236, −57 [−810, −20.7]
*Serum cortisol* (nmol/L) [μg/dL]					
n^a^	18	18	11	5	4
Mean	724 ± 222 [26.2 ± 8.0]	698 ± 161 [25.3 ± 5.8]	559 ± 300 [20.3 ± 10.9]	685 ± 79.0 [24.8 ± 2.9]	628 ± 181 [22.8 ± 6.6]
Range	442, 1,187 [16.0, 43.0]	414, 1,049 [15.0, 38.0]	193, 1,297 [7.0, 47.0]	580, 773 [21.0, 28.0]	414, 856 [15.0, 31.0]
Mean change from core baseline ± SD	–	−26.0 ± 125 [−0.9 ± 4.5]	−151 ± 309 [−5.5 ± 11.2]	−66.1 ± 302 [−2.4 ± 11.0]	−228 ± 283 [−8.3 ± 10.3]
Range		−304, 138 [−11.0, 5.0]	−773, 221 [−28.0, 8.0]	−469, 303 [−17.0, 11.0]	−414, 194 [−15.0, 7.0]
*Plasma ACTH* (pmol/L) [pg/mL]					
n^a^	18	18	11	5	4
Mean ± SD	13.7 ± 11.0 [62.3 ± 50.0]	12.4 ± 8.9 [56.4 ± 40.5]	9.6 ± 4.9 [43.2 ± 22.3]	9.8 ± 3.8 [44.5 ± 17.3]	12.0 ± 6.3 [54.5 ± 28.6]
Range	2, 46 [9.1, 209]	4, 40 [18.2, 182]	4, 17 [18.2, 77.3]	4, 14 [18.2, 63.6]	4, 17 [18.2, 77.3]
Mean change from core baseline ± SD	–	−1.3 ± 7.1 [−5.9 ± 32.3]	−3.4 ± 9.4 [−15.5 ± 42.7]	−3.6 ± 15.2 [−16.4 ± 69.1]	−4.5 ± 8.3 [−20.5 ± 37.7]
Range		−17, 16 [−77.3, 73.0]	−23, 10 [−104, 45.5]	−27, 12 [−122, 54.5]	−14, 5 [−63.6, 22.7]

Core baseline is considered as pre-dose on day 1. Normal range for UFC: 55–276 nmol/24 h (20–100 μg/24 h); normal range for serum cortisol: 221–690 nmol/L (8.0–25.0 μg/dL; 09:00 h measure); normal range for plasma ACTH: <10 pmol/L (<45.5 pg/mL). The mean change from baseline was calculated only in those patients with evaluable measurements at baseline and month 6

^a^n is the number of patients in the primary efficacy population who have a mean UFC value at core baseline and at a particular visit

^b^n = 16

^c^n = 10

#### Serum cortisol and plasma ACTH levels at month 6

Consistent with the reduction in UFC levels, mean serum cortisol (±SD) and plasma ACTH levels decreased from core baseline by −151 ± 309 nmol/L and −3.4 ± 9.4 pmol/L, respectively, at month 6 (Table 1).

### Ongoing effects of pasireotide therapy up to 24 months

The reductions in mean UFC persisted in the four patients who were on study drug at month 24 (Table 1). Of these four patients, three had >50 % reduction in mean UFC from core baseline. One patient had been a responder at the end of the core study. Despite variability in serum cortisol levels, there was an overall reduction in serum cortisol and plasma ACTH relative to core baseline that was maintained throughout the extension (Table 1). There was an overall reduction in body weight and diastolic blood pressure over time (Table 2). Clinical improvements were not limited to patients who achieved UFC control. The sample size was not large enough to assess the significance of these results.

**Table 2:** Change in vital signs from core baseline to day 15 and months 6, 12 and 24

	Core baseline	Day 15	Month 6	Month 12	Month 24
*Body weight* (kg)					
n	19	19	12	7	4
Mean ± SD (range)	81.7 ± 19.3 (58.8, 132.4)	80.1 ± 19.7 (56.5, 132.5)	74.3 ± 11.5 (46.0, 90.8)	73.1 ± 14.0 (49.0, 91.7)	71.9 ± 17.0 (48.3, 88.5)
Mean percentage change from core baseline ± SD (range)	–	−2.0 ± 4.1 (−15.5, 5.3)	−6.9 ± 9.5 (−25.1, 7.3)	−11.2 ± 10.8 (−23.8, 4.4)	−11.1 ± 12.3 (−20.8, 6.2)
*Systolic blood pressure* (mmHg)					
n	19	19	12	7	4
Mean ± SD (range)	129.8 ± 13.7 (110, 155)	127.7 ± 11.4 (110, 146)	120.9 ± 8.9 (100, 131)	129.1 ± 10.9 (115, 145)	129.0 ± 16.4 (110, 145)
Mean percentage change from core baseline ± SD (range)	–	−1.1 ± 8.4 (−17.9, 13.3)	−2.6 ± 7.4 (−16.7, 8.7)	1.2 ± 8.6 (−10.2, 12.5)	−0.1 ± 15.0 (−12.9, 20.8)
*Diastolic blood pressure* (mmHg)					
n	19	19	12	7	4
Mean ± SD (range)	85.5 ± 8.1 (70, 100)	81.7 ± 9.9 (60, 102)	77.6 ± 8.3 (60, 90)	74.9 ± 12.1 (53, 90)	79.3 ± 16.4 (60, 100)
Mean percentage change from core baseline ± SD (range)	–	−3.9 ± 12.3 (−25.0, 22.9)	−7.4 ± 8.4 (−25.0, 2.9)	−14.1 ± 11.7 (−36.1, 2.4)	−6.52 ± 19.2 (−25.0, 20.5)

### Pharmacokinetics

Mean ± SD C_trough_ levels at month 6 were 17.4 ± 11.6 and 19.1 ± 10.9 μg/L in UFC responders (n = 4) and reducers (n = 6), respectively, and 5.2 μg/L in non-reducers (n = 1). These values were higher than those at the end of the core study, when mean ± SD C_trough_ levels were 7.0 ± 4.3 and 4.4 ± 1.1 μg/L in UFC responders and reducers, respectively, and 2.7 μg/L in non-reducers.

### Safety and tolerability of pasireotide up to month 24

All patients experienced at least one AE during the study. With the exception of hyperglycemia, AEs were generally as expected and were mostly related to transient gastrointestinal discomfort (Table 3). One patient discontinued treatment because of an AE (new-onset type 2 diabetes mellitus). Thirteen patients reported a hyperglycemia-related adverse event: 11 patients had a reported AE of hyperglycemia, one patient had increased blood glucose, and one patient reported type 2 diabetes. Two patients had grade 4 AEs: one was increased γ-glutamyltransferase levels and the other was type 2 diabetes mellitus. Both events were suspected by the investigator to be related to study drug. No patients died during the study.

**Table 3:** Most frequently occurring adverse events (>5 patients) regardless of study-drug relationship

Adverse event	n (%)
Diarrhea	13 (68.4)
Nausea	12 (63.2)
Hyperglycemia	11 (57.9)
Abdominal pain	9 (47.4)
Headache	7 (36.8)
Injection-site pain	6 (31.6)
Dizziness	5 (26.3)
Fatigue	5 (26.3)
Injection-site pruritus	5 (26.3)

At extension baseline, eight of the 19 patients had HbA_1c_ values available, all of which were <ULN (extension baseline mean ± SD: 5.78 ± 0.51 %). HbA_1c_ was not measured during the core study. Six patients had post-baseline HbA_1c_ levels shifted to a higher category at some point during the extension and were treated with modification of diet or with antidiabetic medication, including metformin, glimepiride, exenatide, pioglitazone or insulin. Five of these six patients remained at a higher post-baseline HbA_1c_ level at their last assessment.

All patients experienced an increase in fasting plasma glucose (FPG) level at some point during the extension study (see Supplementary Material). Of the 16 patients with normal FPG at extension baseline, FPG levels shifted to between 100 and <126 mg/dL (5.6 to <7.0 mmol/L) in six patients, between 126 and <200 mg/dL (7.0 to <11.1 mmol/L) in four patients and ≥200 mg/dL (≥11.1 mmol/L) in six patients at some point during the extension study. All patients had improved FPG levels at their last assessment.

Electrocardiogram intervals were generally within normal ranges during the study. One patient had a newly occurring QT of 450–480 ms, another patient had a newly occurring QTcB of 450–480 ms, and one patient had a QRS interval of >110 ms (not clinically significant).

## Discussion

The results of this study support the extended treatment with pasireotide in some patients with Cushing’s disease. After 6 months of therapy, 10 of 18 patients (56 %) achieved reduced UFC levels compared with core baseline, and four patients (22 %) had normalized UFC levels. Mean serum cortisol and plasma ACTH levels also decreased over this period, irrespective of whether UFC control was achieved. These pasireotide-induced reductions were maintained in four patients who were treated for over 24 months. These findings led to the initiation of a Phase III study of pasireotide in 162 patients with persistent/recurrent or de novo Cushing’s disease [13]. The Phase III study found that pasireotide treatment led to a rapid and sustained decrease in UFC levels. The UFC normalization rate at month 6 in the current study (22 %) and the Phase III study (20.4 %) was similar. Based on the findings from the Phase III study, pasireotide was approved in the EU and US for the treatment of patients with Cushing’s disease for whom surgery is not an option or who decline surgery. The current study shows that this response can be maintained for at least 24 months in some patients. In fact, a patient case from this study has illustrated long-term UFC control without escape or serious AEs during >7 years of treatment with pasireotide sc [14].

Long-term reductions in body weight and diastolic blood pressure were found in the current study, which in part may reflect the sustained reduction in cortisol levels and may attenuate the considerable morbidity observed in patients with Cushing’s disease [2–4]. While the sample size in the present study was not large enough to test the statistical significance of this result, significant improvements in clinical signs and symptoms were observed in the much larger Phase III study of pasireotide in Cushing’s disease [13, 15].

The results of the current study suggest that there may be a relationship between the plasma concentration of pasireotide and its effect on UFC levels. Interestingly, trough plasma concentrations of pasireotide were higher at month 6 than at the end of the core study. The reasons for this are unclear, and it is difficult to fully interpret this finding because of the small sample size.

The safety profile of pasireotide in this study is similar to that of previous pasireotide studies [11, 13]. In line with the safety profile of other somatostatin analogues, the most frequently occurring drug-related AEs are mild-to-moderate and transient gastrointestinal disturbances. The prevalence of hyperglycemia-related AEs in this study was higher than seen with other somatostatin analogues in other indications [16–18]. Patients with Cushing’s disease are predisposed to alterations in glucose homeostasis [19], and a higher prevalence of diabetes mellitus and glucose intolerance has been reported in patients with Cushing’s disease compared with the general population [20]. In the present study, none of the eight patients with available glycemic data had elevated HbA_1c_ levels at extension baseline. However, the possibility of glucose intolerance could not be excluded in these patients. During the treatment period, hyperglycemia-related AEs were reported in 68 % of patients (13/19). Seven continued pasireotide treatment with concomitant treatment for hyperglycemia-related AEs, five continued with no medication for hyperglycemia and one patient discontinued. It is therefore important to monitor glucose levels during pasireotide treatment and initiate prompt medical intervention and close follow-up [21].

There are several limitations of this study that may have affected the findings, most notably the small number of patients who entered the extension period and who continued to receive pasireotide after 6 months. This limits the conclusions that can be drawn from some of the analyses, particularly of the pharmacokinetic data. In addition, HbA_1c_ measurements were available for only 42 % of patients at baseline, which rendered the study less informative than planned with regard to the effects of pasireotide on glucose homeostasis. However, the results of the study are consistent with the much larger Phase III study of pasireotide [13].

In conclusion, pasireotide offers a tumor-directed medical therapy and may be effective for the long-term treatment of some patients with Cushing’s disease.

## Electronic supplementary material

Below is the link to the electronic supplementary material.

Supplementary material (DOCX 32 kb)
