# Supplementary material for: Extended treatment of Cushing’s disease with pasireotide: results from a 2-year, Phase II study
Source: Pituitary. 2013 Aug 14;17(4):320–6. doi: 10.1007/s11102-013-0503-3 (PMC4085509; doi:10.1007/s11102-013-0503-3)
Supplement: Supplementary file 1 — Supplementary material (DOCX 32 kb) [file 11102_2013_503_MOESM1_ESM.docx]

**Supplementary Material**

**Extended treatment of Cushing’s disease with pasireotide:
results from a 2-year, Phase II study**

*Pituitary* manuscript PITU1113

**Table of contents**

| Patients | 2 |
| --- | --- |
| Inclusion criteria | 2 |
| Exclusion criteria | 2 |
| Treatment interval | 3 |
| Study design | 3 |
| Assay details | 4 |
| Effects of pasireotide treatment on UFC, serum cortisol and plasma ACTH at month 6 | 4 |
| Changes in HbA_1c_ and fasting plasma glucose | 7 |

**Patients**

In the core study, Cushing’s disease was confirmed by: 1) mean of two consecutive 24-hour urinary free cortisol (UFC) levels at least twice the upper limit of normal (ULN), as measured by the local laboratory at each participating center; 2) morning plasma adrenocorticotropic hormone (ACTH) levels within or above the normal range; and
3) either confirmation by magnetic resonance imaging of a pituitary macroadenoma (≥1 cm) or an inferior petrosal sinus/peripheral gradient of ACTH >3 after CRH stimulation [1].

**Inclusion criteria**

Patients were included if they did not experience any unacceptable adverse events (AEs) or tolerability issues during the original 15-day treatment period. Female patients of childbearing potential who had not undergone clinically documented total hysterectomy and/or ovariectomy, or tubal ligation, had to agree to use barrier contraception throughout the course of the extension study and for 1 month after the study had ended.

**Exclusion criteria**

Patients could not enrol in the extension study if they experienced any of the following during the core study: development of poorly controlled diabetes mellitus (as indicated by ketoacidosis or HbA_1c_ >10%); persistent alanine aminotransferase/aspartate aminotransferase or alkaline phosphatase levels >2.5 x ULN; serum creatinine >2.0 x ULN and/or serum bilirubin >2 x ULN; abnormal coagulation (prothrombin time and partial thromboplastin time elevated by 30% above normal limits) or white blood cell (<3.0 x 10^9^/L), hemoglobin (<12.0 g/dL for females, <13.0 g/dL for males) or platelet count (<100 x 10^9^/L); or any other unacceptable AEs or tolerability problems.

**Treatment interval**

The eligibility of patients to continue treatment with pasireotide as part of the extension study was determined when the final results of the core study were available. During this interim period, patients did not receive pasireotide. Of the 18 patients in the primary efficacy analysis, seven had a treatment interval of more than 30 days between the end of the core phase and the start of the extension phase (range: 41–367 days), three had a treatment interval of less than 30 days, and eight had no treatment interval.

**Study design**

Patient visits occurred every 2 weeks for the first 3 months and then every 3 months for assessments of UFC, serum cortisol, plasma ACTH, hematology, blood chemistry, HbA_1c_, electrocardiogram (ECG) and vital signs. Patients were referred to a diabetes specialist for evaluation and appropriate management if any of the following were present:

- No prior history of impaired fasting blood glucose or diabetes mellitus but experience of fasting plasma glucose (FPG) >240 mg/dL (repeated and confirmed after 2 weeks) during the study
- Prior history of FPG >240 mg/dL or diabetes mellitus and FPG remained poorly controlled and/or with two consecutive HbA_1c_ measurements >8% but <10%
- HbA_1c_ ≥10% at any time during the study

**Assay details**

UFC levels were measured with two 24-hour urine specimens collected in the 48 hours prior to core baseline and at study visits during the extension study. Serum cortisol was measured with electrochemiluminescence immunoassay (ECLIA), using the Elecsys^®^ cortisol reagent kit (Roche Diagnostics, Indianapolis, IN, USA), which has a lower limit of quantification (LLOQ) of 0.05 nmol/L (0.018 μg/dL) and intra- and inter-assay coefficients of variation of 1.2% and 1.4%, respectively. UFC levels were measured with ECLIA, using the Elecsys^®^ cortisol reagent kit with prior dichloromethane extraction to reduce the amount of cortisol metabolites and conjugates.

Plasma ACTH was assayed with the Immulite^®^ 2000 ACTH kit (DPC, Los Angeles, CA, USA), which has a lower limit of detection of 9 pg/mL (1.98 pmol/L) and intra- and inter-assay coefficients of variation of 7.7% and 8.5%, respectively. Pasireotide plasma concentrations were measured using a validated radioimmunoassay with an LLOQ of 150 pg/mL.

**Effects of pasireotide treatment on UFC, serum cortisol and plasma ACTH at month 6**

Eleven patients remained on pasireotide 600 µg sc bid during the extension; one patient had a dose decrease to 450 µg sc bid and subsequent increase back to 600 µg. Seven patients had a dose increase to pasireotide 900 µg sc bid at some point before month 6. Of the 18 patients included in the primary efficacy analysis, four responders had a decrease in mean UFC from core baseline to month 6 (Supplementary Table 1). Two of the four UFC responders at month 6 were taking pasireotide 600 μg sc bid, one was taking pasireotide 300 μg sc bid, and one had been uptitrated to 900 μg sc bid. The six reducers had a decrease in mean UFC from core baseline to month 6. The one non-reducer with evaluable UFC measurements at month 6 had an increase in mean UFC from core baseline to month 6.

Similarly, patients who were classified as UFC responders and UFC reducers had a decrease in mean serum cortisol from core baseline to month 6, respectively. The one UFC non-reducer had increased mean serum cortisol from core baseline to month 6.

Patients from all three subgroups had a decrease in mean plasma ACTH from core baseline to month 6 of the extension.

**Supplementary Table 1. Summary and change from baseline to month 6 in mean UFC, serum cortisol and plasma ACTH by UFC response subgroup**

|  | **n** | **Mean ± SD** | **Change from core baseline** |
| --- | --- | --- | --- |
| **Mean UFC (nmol/24h) [µg/24h]** | | | |
| *UFC responders*  Core baseline  Month 6 | 3  4^a^ | 1516 ± 902 [549 ± 327]  181 ± 28 [66 ± 10] | **–**  -1330 ± 922^b^ [-549 ± 327] |
| *UFC reducers*  Core baseline  Month 6 | 6  6 | 1243 ± 952 [450 ± 345]  566 ± 397 [205 ± 118] | **–**  -677 ± 736 [-245 ± 283] |
| *UFC non-reducers*  Core baseline  Month 6 | 8  1 | 1091 ± 599 [395 ± 217]  506 [183] | **–**  34.5^c^ [12.5] |
| **Mean serum cortisol (nmol/L) [µg/dL]** | | | |
| *UFC responders*  Core baseline  Month 6 | 4  4 | 849 ± 203 [30.8 ± 7.4]  655 ± 428 [23.7 ± 15.5] | **–**  -193 ± 318 [-7.0 ± 11.5] |
| *UFC reducers*  Core baseline  Month 6 | 6  6 | 653 ± 214 [23.7 ± 7.8]  478 ± 230 [17.3 ± 8.3] | **–**  -175 ± 329 [-6.3 ± 11.9] |
| *UFC non-reducers*  Core baseline  Month 6 | 8  1 | 714 ± 236 [25.9 ± 8.6]  662 [24.0] | **–**  166^c^ [6.0] |
| **Mean plasma ACTH (pmol/L) [pg/mL]** | | | |
| *UFC responders*  Core baseline  Month 6 | 4  4 | 9.8 ± 5.7 [44.5 ± 25.9]  6.8 ± 4.2 [30.9 ± 19.1] | **–**  -3.0 ± 7.9 [-13.6 ± 35.9] |
| *UFC reducers*  Core baseline  Month 6 | 6  6 | 14.2 ± 10.7 [64.5 ± 48.6]  10.2 ± 4.4 [46.4 ± 20.0] | **–**  -4.0 ± 11.8 [-18.2 ± 53.6] |
| *UFC non-reducers*  Core baseline  Month 6 | 8  1 | 15.4 ± 13.5 [70.0 ± 61.4]  17.0 [77.3] | **–**  -1.0^c^ [-4.5] |

^a^One of the four responders at month 6 had only one UFC value at baseline and is not included in the baseline values.

^b^Based on n=3.

^c^Change from baseline in the one patient with values at both time points.

Note: Core baseline is considered as pre-dose on day 1. Normal range for UFC: 55–276 nmol/24h (20–100 µg/24h); normal range for serum cortisol: 221–690 nmol/L (8.0–25.0 µg/dL; 09:00 h measure); normal range for plasma ACTH: <10 pmol/L (<45.5 pg/mL). The mean change from baseline was calculated only in those patients with evaluable measurements at baseline and month 6.

**Changes in HbA_1c_ and fasting plasma glucose**

During treatment in the extension, there was a mean increase in HbA_1c_ from extension baseline to last available visit of 0.91 ± 0.84%. Six patients had post-baseline HbA_1c_ levels shifted to a higher category at some point during the extension study; of these, three patients had HbA_1c_ levels <7%, and three patients had levels between 7% and 9%. There were no shifts to levels of HbA_1c_ >9%. Five of these six patients remained at a higher post-baseline HbA_1c_ level at their last assessment; of these, four patients had levels shifted to >ULN but <7%, and one patient had levels shifted to between 7% and 9%.

Mean FPG increased from core baseline to month 6 in patients receiving pasireotide sc 600 and 900 µg bid sc (Supplementary Table 2). All patients had improved FPG levels at their last assessment: six patients had normal FPG levels, three had FPG between 100 and <126 mg/dL (5.6 to <7.0 mmol/L), four had FPG between 126 and <200 mg/dL (7.0 to 11.1 mmol/L), and three had FPG ≥200 mg/dL (≥11.1 mmol/L).

**Supplementary Table 2. Fasting plasma glucose levels in the safety population (n=19) at core baseline, day 15, and extension study months 3, 6, 12 and 24**

|  | **Pasireotide dose** | |
| --- | --- | --- |
| **Glucose (mg/dL)** | **600 µg bid** | **900 µg bid** |
| **Core baseline**  n  Mean ± SD | 19  94 ± 40 | 7  115 ± 59 |
| **Day 15**  n  Mean ± SD  Mean change from core baseline ± SD | 18  121 ± 50  27 ± 38 | –  –  – |
| **Month 3**  n  Mean ± SD  Mean change from core baseline ± SD | 9  178 ± 68  98 ± 71 | 5  167 ± 105  39 ± 106 |
| **Month 6**  n  Mean ± SD  Mean change from core baseline ± SD | 5  184 ± 60  108 ± 62 | 4  174 ± 76  38 ± 44 |
| **Month 12**  n  Mean ± SD  Mean change from core baseline ± SD | 2  181 ± 23  117 ± 15 | 3  98 ± 48  -3 ± 77 |
| **Month 24**  n  Mean ± SD  Mean change from core baseline ± SD | 2  134 ± 40  53 ± 71 | 2  107 ± 18  -4.0 ± 57 |

**Reference**

1. Boscaro M, Ludlam WH, Atkinson B, Glusman JE, Petersenn S, Reincke M, Snyder P, Tabarin A, Biller BM, Findling J, Melmed S, Darby CH, Hu K, Wang Y, Freda PU, Grossman AB, Frohman LA, Bertherat J (2009) Treatment of pituitary dependent Cushing's disease with the multi-receptor ligand somatostatin analog pasireotide (SOM230): a multicenter, phase II trial. J Clin Endocrinol Metab 94:115-122.
